# Professional development for clerkship administrators: a 16-year overview of the clerkship administrator certificate program

**DOI:** 10.1080/10872981.2019.1710327

**Published:** 2019-12-31

**Authors:** Donnita Pelser, Cathy Chavez, Lindsey Allison, Virginia Cleppe, Gary L. Beck Dallaghan

**Affiliations:** a Department of Pediatrics, University of Kansas School of Medicine-Wichita, Wichita, KS, USA; b Carver College of Medicine, University of Iowa Stead Family Children’s Hospital, Iowa City, IA, USA; c Office of Medical Education, Wright State University Boonshoft School of Medicine, Dayton, OH, USA; d Department of Pediatrics, The Medical College of Wisconsin, Milwaukee, WI, USA; e Office of Medical Education, The University of North Carolina School of Medicine, Chapel Hill, NC, USA

**Keywords:** Medical student education, clerkship, clerkship coordinator, clerkship administrator, professional development

## Abstract

Background: Increasing accreditation requirements as well as transformations in medical school curricula necessitate administrative staff who are not only invested in the clerkship coordinator role but also view what they do as a career. To date, there has been a lack of professional development opportunities for clerkship administrators. Methods: In 2003, the Central Group on Educational Affairs of the Association of American Medical Colleges recognized a need for professional development for clerkship administrators. The Clerkship Administrator Certificate Program emerged from that decision and presented for the first time in 2004 in Omaha, Nebraska. This article provides an overview of the program, how it has been evaluated, and how it continues to evolve. Results: The program had two guiding principles: to offer professional development opportunities for clerkship administrators through interactive workshops and to ensure the program was feasible both in terms of completion and in cost. Over the past 16 years, the Clerkship Administrator Certificate Program workshops have been delivered to over 300 clerkship administrators. Of those, 206 have completed a project in order to receive their certificate. Projects have related to innovations in medical education (n = 41), grading (n = 26), professional development (n = 26), and patient care (n = 20) to name a few. Discussion: In order to meet the demands for presenting the workshops, a train-the-trainer model has been employed to expand the number of individuals presenting the workshops. Additional research needs to be done to determine influence of the program on future professional development endeavors.

## Introduction

Over the past 15 years, the role of the administrative staff supporting required clinical medical student experiences has evolved. No longer can a clerkship director rely on a part-time staff person to aid with the daily challenges presented during a clerkship. In 2003, the Alliance for Clinical Education published a position statement asserting a full-time clerkship coordinator is necessary to effectively run a clinical clerkship [1]. Subsequently, the fifth edition of The Guidebook for Clerkship Directors provided clear examples of how clerkship coordinators complement the educational mission in ways never before considered [2].

As with residency program coordinators [3], increasing accreditation requirements along with transformations in medical school curricula necessitate administrative staff who embrace the clerkship coordinator role as a profession [4,5]. Because higher level skills are needed to manage clinical clerkships, we will henceforth use the term ‘clerkship administrator’ to reflect the skills and competencies of this profession.

Continuous professional development is essential for clerkship administrators to appropriately manage the medical student education mission as well as collect data for accreditation of the medical school. Residency program coordinators also have opportunities for professional development [6] through their specialty-specific residency organizations. Clerkship administrators have had limited opportunities unless their specialty-specific clerkship organization offered workshops targeted for them.

In 2003, the executive committee of the Central Group on Educational Affairs (CGEA) of the Association of American Medical Colleges recognized a need for professional development for clerkship administrators. The leaders noted that this population was absent from meetings and wanted to offer a program specifically for them. The CGEA Clerkship Administrator Certificate Program emerged from that decision and presented for the first time in 2004 in Omaha, Nebraska. This article provides an overview of the program, how it has been evaluated, and how it continues to evolve.

## Methods

### Certificate program content

The development of this program had two guiding principles. The first was to elevate clerkship administrators as medical education professionals through workshops and individual projects. The second was to offer something feasible, both in terms of completion and in cost. At the time this program was conceived in 2003, many medical schools did not have professional development funds allocated for clerkship administrators to attend meetings. We therefore encapsulated the required workshops into a 4-h pre-conference block. This would allow participants to complete the core material during one meeting.

The content for the program was adapted from a faculty development leadership program offered at one of the author’s (GBD) home institution. The philosophy behind the sessions chosen was to encourage clerkship administrators to embrace their role as an educational leader. Although they may not be content experts in clinical medicine, their skills in other domains are equally valuable to medical student education. The first workshop walked participants through the importance of having a personal vision and mission. As part of that discussion, participants identified personal and professional responsibilities, identifying which are personal priorities and perceived priorities of the institution. The objectives of this session included: 1) Articulate your personal mission; 2) Correlate your passions with your mission; and 3) Compare and contrast your personal mission, vision and values with that of your organization.

The second workshop focused on leadership skills. Using the Good to Great framework [7], levels of leadership were addressed to identify where participants felt they fit in the five levels of leadership, which included executive leader (level 5) to highly capable individual (level 1). Association of these leadership levels was mapped to the mission and vision discussions to demonstrate how these concepts flow together. Using the list of personal and professional responsibilities, participants were able to identify how some duties relate to executive leadership while some require a highly capable individual. The objectives of this session included: 1) Identify the level at which you are a leader in medical education and 2) Compare your mission with the core purpose of your clerkship.

These two workshops have been core to the Clerkship Administrator Certificate Program since its inception. During the third year the program was offered at the CGEA meeting, a session was added to train participants about the importance of emotional intelligence and how to approach difficult conversations. One of the first graduates of the certificate program proposed this session and joined the team of facilitators. Research related to the importance of emotional intelligence was presented, asking participants to reflect on the material in small groups. Using the framework for crucial conversations [8], multiple scenarios provided opportunities to strategize how to address issues in a respectful manner. In recent years, participants take the Myers–Briggs Type Indicator [9] to better understand their own personality type better, which has complemented the discussions about emotional intelligence and communication skills. The objectives of this session included: 1) Describe the importance of emotional intelligence and its role in leadership and 2) Develop strategies for using emotional intelligence to achieve desired outcomes in critical conversations.

In order to obtain the certificate of completion, participants must complete a project over the course of a year, or longer. Offering workshops contribute to continuing education, but often this information is not applied after a meeting. Since our first goal was to elevate the role of clerkship administrators as medical education professionals, we included the project requirement to apply leadership through pursuit of educational research or quality improvement. Examples of projects were discussed so participants understood what could be done. The expectation was that participants would return the following year to present their work for new attendees. Flexibility was also built into this requirement, taking into consideration institutional budgets, by allowing a written report summarizing the project and results be submitted in lieu of a presentation.

### Program evaluation

This program has been evaluated in two ways. The first has been through completion of an evaluation form at the end of each program. The second was from completion of a project.

Program evaluations have been revised and modified over the past 15 years. Initially, standard program evaluations from the CGEA meeting were used. As workshops were presented at other national meetings, such as the Council on Medical Student Education in Pediatrics (COMSEP) and the Association of Directors of Medical Student Education in Psychiatry (ADMSEP), their standard evaluations were collected to assess the workshops.

## Results

**Figure 1.**
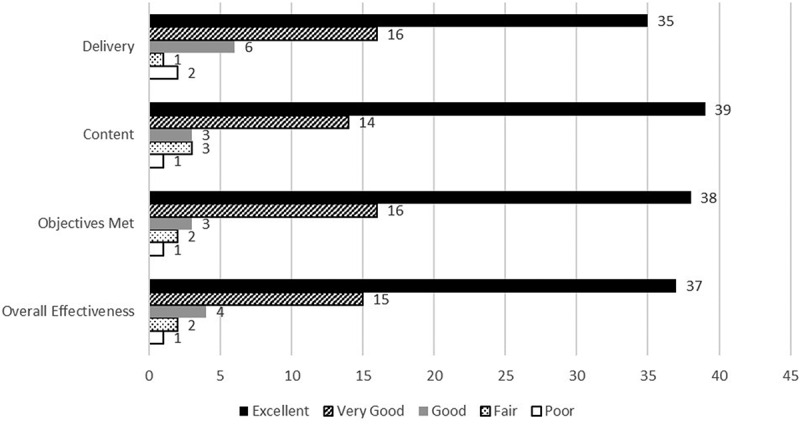
COMSEP program evaluations from 2012 to 2017. Standard evaluations for workshops for COMSEP include delivery, content, objectives, overall workshop effectiveness. This figure summarizes evaluations obtained from 2012 to 2017. Frequency of responses is included for each item on the evaluation

**Figure 2.**
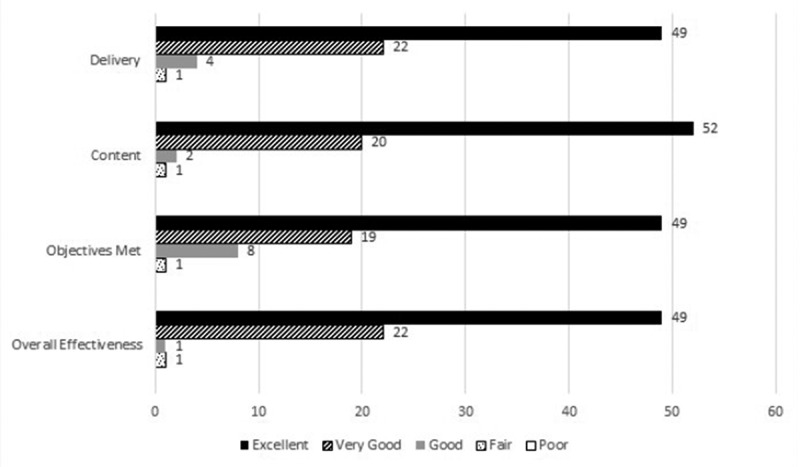
Evaluations from invited presentations (2014–2016). Evaluations were obtained from presenting the program in Arizona, Nebraska and Virginia. Frequency of responses is included for each item on the evaluation

As presented in Figures 1 and 2, participants have regularly provided the workshops with high ratings. Narrative comments on the workshop evaluations have been reviewed to continuously improve the sessions. As a result of these comments, additional material has been added, such as a discussion about the Liaison Committee on Medical Education (LCME) elements relevant to clerkships and the role clerkship administrators have in meeting those standards. The information dovetails with leadership roles and having crucial conversations with faculty, staff, and students about accreditation requirements.

### Projects completed

To receive a certificate, participants had to complete a project once they completed the workshops. Projects took the form of quality improvement or educational research. After the first year of the program, clerkship director names and emails were obtained so the certificate program leaders could contact them. We explained to the clerkship director the project completion requirement and encouraged the clerkship director to work with the clerkship administrator to undertake a project mutually beneficial for their clerkship.

To date, 206 projects have been completed. Several of these projects have been submitted through the CGEA, COMSEP or ADMSEP peer-review process as poster presentations at the annual meetings. Table 1 lists the main topics of these projects in order of frequency, with the most frequent topic first. Innovations in medical education were the most common projects. Other projects dealt with patient care, professional development, and examinations. These projects reflect some of the essential skills Steckelman et al. noted residency coordinators need as medical education evolves[10].

**Table 1.** Project themes and exemplar titles

Project Theme	No.	Exemplar Title
Innovations	41	The Concise CRISIS Management Guide for Medical Students
Professional Development	26	Boot Scootin’ Boogie Collaboration to Achieve Success in Both Resident and Student Education
Grading	26	Implementation of the OB/GYN Grading Program Weighing the Benefits
Patient Care	20	Best Practices – Patient Presentations in Surgery Clerkship
Learning Environment	18	Mixed Methods: Evaluation of Inpatient Learning Environment in Two Hospitals
Examinations	17	Analyzing NBME Minimum Passing Scores when Students do CLIPPcases
Orientation	15	Enhancing the Clerkship Orientation Experience
Professionalism	11	Professionalism Beyond the White Coat
Accreditation	10	LCME Standards and the Clerkship Administrator
Career Choice	6	Telling Your Story to Expand Your Professional Opportunities: Recognizing the Steps Needed for Improved Proficiency and Career Enhancement or Promotion
House Officer Evaluations	6	Collaboration of the Interdisciplinary Team
Visiting Students	2	Credentialing and Other Necessary Evils

A number of programs have invited the authors to deliver the certificate program at their own institutions, providing further evidence that the program is feasible, affordable, and valued. The University of Iowa was the first medical school to invite us to present to all of their educational administrators. The certificate program leaders worked with faculty at the institutions to ensure projects were completed and either presented locally or a written summary was submitted. Over the years, the certificate program has been presented in Arizona, California, Florida, Illinois, Iowa, Kansas, Maryland, Missouri, Nebraska, Texas, Virginia, and Vermont.

## Building on existing literature

Over the past 16 years, we have had the opportunity to offer the CGEA Clerkship Administrator Certificate Program workshops to over 300 clerkship administrators. Of those, 206 have completed a project in order to receive their certificate. The continued invitations to offer the program at institutions as well as the expansion to specialty-specific organizations demonstrate the need for professional development programs for clerkship administrators.

Our guiding principles for the program continue to be relevant. Medical schools are facing fiscal challenges with the changing health-care landscape[11]. Therefore, providing a cost-effective professional development program to individuals vital to clinical medical education is essential[12]. The process of continuous quality improvement with the program content has allowed the workshops to educate clerkship administrators about personal and career-specific topics.

The authors learned a number of lessons early, including first and foremost the need for flexibility with project completion requirements. Initially, our hope in requiring a presentation was that it would encourage schools to invest in clerkship administrators’ careers and allocate funds to attend at least one meeting per year. However, this was proven to be unrealistic and led to alternative methods of presentations such as permitting local oral presentations or submitting a structured written report.

Additionally, the architects of this program later recognized the need for succession planning. As they moved out of their roles as clerkship administrators (GBD became an assistant dean, VC became residency program manager), it was evident that a train-the-trainers model needed to be adopted. When the current program leaders took over, they expanded the team of individuals who are trained to deliver the core content to ensure that if a presenter was unable to attend, others could step in to deliver the content.

A limitation of this report is the lack of follow-up data regarding the long-term impact of this program on job satisfaction or continuing professional development of the participants. Although the impact has been noticeable through increased presence and formal programming for clerkship administrators at ADMSEP, CGEA and COMSEP meetings, the true magnitude has not been investigated. A future endeavor is to conduct follow-up studies with participants to inquire what sort of catalyst effect the certificate program has had for them.

## Conclusions

As accreditation requirements in medical student education expand and health-care delivery becomes more complex, clerkship directors need clerkship administrators capable of managing the educational program. Clerkship administrators need to understand their role in the continuum of medical student education. The Clerkship Administrator Certificate Program has proven to be a robust, affordable professional development program for clerkship administrators. After 16 years and over 200 completed projects, the need for dedicated professional development for clerkship administrators continues.

## Data Availability

Data are available by contacting the corresponding author.

## Inclusion Criteria

All of the authors developed the initial plan for this manuscript and have participated in all aspects of writing.

## Attestation

Ms. Pelser, Ms. Chavez, Ms. Allison, Ms. Cleppe, and Dr. Beck Dallaghan have given final approval of this manuscript for publication.

## References

[1] Pangaro L, Fincher RM, Bachicha J, et al. Expectations of and for clerkship directors: a collaborative statement from the Alliance for clinical education. Teach Learn Med. 2003;15(3):217–5. 12855395 10.1207/S15328015TLM1503_12

[2] Allison L, Pelser D, Meyer ME. Vital roles the clerkship administrator plays in medical student education. In: Morgenstern BZ, Horak H, Konopasek L, et al., editors. Alliance for clinical education guidebook for clerkship directors. 5th ed. North Syracuse, NY: Gegensatz Press; 2019;33–48.

[3] Grant RE, Murphy LA, Murphy JE. Expansion of the coordinator role in orthopaedic residency program management. Clin Orthop Relat Res. 2008;466:737–742. 18196362 10.1007/s11999-007-0110-6 PMC2505208

[4] Beck GL, Cleppe V. Team effort: the role of the clerkship administrator and professional opportunities for them. In: Morgenstern BZ, editor. The guidebook for clerkship directors. 4th ed. Syracuse, NY: Gegensatz Press; 2012;475–488.

[5] Beck GL, Cleppe V, Marts L, et al. Redefining the role of clerkship administrator. In: Fincher RE, editor. The guidebook for clerkship directors. 3rd ed. Omaha, NE: Alliance for Clinical Education; 2005;365–377.

[6] O’Sullivan PS, Heard JK, Petty M, et al. Educational development program for residency program directors and coordinators. Teach Learn Med. 2006;18(2):142–149. 16626273 10.1207/s15328015tlm1802_9

[7] Collins J. Good to great: why some companies make the leap … and others don’t. New York, NY: HarperCollins Publishers; 2001.

[8] Patterson K, Grenny J, McMillan R, et al. Crucial conversations: tools for talking when stakes are high. Second ed. New York, NY: McGraw-Hill Education; 2011.

[9] The Myers & Briggs Foundation. Take the MBTI instrument; [cited 2019 1118]. Available from: https://www.myersbriggs.org/my-mbti-personality-type/take-the-mbti-instrument/

[10] Stuckelman J, Zavatchen S, Jones SA. The evolving role of the program coordinator: five essential skills for the coordinator toolbox. Acad Radiol. 2017;24:725–729. 28262520 10.1016/j.acra.2016.12.021

[11] Kerschner JE, Hedges JR, Antman K, et al. Recommendations to sustain the academic mission ecosystem at U.S. medical schools. Acad Med. 2018;93(7):985–989. 29538107 10.1097/ACM.0000000000002212 PMC6019606

[12] Dubois L, Marsh T, Demers LB. Program coordinator professional development: definition, perception of importance, motivating factors, and barriers. Am J Med. 2019;132(1):114–118. 30240687 10.1016/j.amjmed.2018.09.001

